# Role of Glycolysis and Fatty Acid Synthesis in the Activation and T Cell-Modulating Potential of Dendritic Cells Stimulated with a TLR5-Ligand Allergen Fusion Protein

**DOI:** 10.3390/ijms232012695

**Published:** 2022-10-21

**Authors:** Alexandra Goretzki, Yen-Ju Lin, Jennifer Zimmermann, Hannah Rainer, Ann-Christine Junker, Sonja Wolfheimer, Stefan Vieths, Stephan Scheurer, Stefan Schülke

**Affiliations:** Molecular Allergology, Paul-Ehrlich-Institut, Paul-Ehrlich-Straße 51–59, 63225 Langen, Germany

**Keywords:** immune metabolism, Warburg, fusion protein, allergy, flagellin, Bet v 1

## Abstract

Trained immune responses, based on metabolic and epigenetic changes in innate immune cells, are de facto innate immune memory and, therefore, are of great interest in vaccine development. In previous studies, the recombinant fusion protein rFlaA:Betv1, combining the adjuvant and toll-like receptor (TLR)5-ligand flagellin (FlaA) and the major birch pollen allergen Bet v 1 into a single molecule, significantly suppressed allergic sensitization in vivo while also changing the metabolism of myeloid dendritic cells (mDCs). Within this study, the immune–metabolic effects of rFlaA:Betv1 during mDC activation were elucidated. In line with results for other well-characterized TLR-ligands, rFlaA:Betv1 increased glycolysis while suppressing oxidative phosphorylation to different extents, making rFlaA:Betv1 a suitable model to study the immune–metabolic effects of TLR-adjuvanted vaccines. In vitro pretreatment of mDCs with cerulenin (inhibitor of fatty acid biosynthesis) led to a decrease in both rFlaA:Betv1-induced anti-inflammatory cytokine Interleukin (IL) 10 and T helper cell type (TH) 1-related cytokine IL-12p70, while the pro-inflammatory cytokine IL 1β was unaffected. Interestingly, pretreatment with the glutaminase inhibitor BPTES resulted in an increase in IL-1β, but decreased IL-12p70 secretion while leaving IL-10 unchanged. Inhibition of the glycolytic enzyme hexokinase-2 by 2-deoxyglucose led to a decrease in all investigated cytokines (IL-10, IL-12p70, and IL-1β). Inhibitors of mitochondrial respiration had no effect on rFlaA:Betv1-induced IL-10 level, but either enhanced the secretion of IL-1β (oligomycin) or decreased IL-12p70 (antimycin A). In extracellular flux measurements, mDCs showed a strongly enhanced glycolysis after rFlaA:Betv1 stimulation, which was slightly increased after respiratory shutdown using antimycin A. rFlaA:Betv1-stimulated mDCs secreted directly antimicrobial substances in a mTOR- and fatty acid metabolism-dependent manner. In co-cultures of rFlaA:Betv1-stimulated mDCs with CD4+ T cells, the suppression of Bet v 1-specific TH2 responses was shown to depend on fatty acid synthesis. The effector function of rFlaA:Betv1-activated mDCs mainly relies on glycolysis, with fatty acid synthesis also significantly contributing to rFlaA:Betv1-mediated cytokine secretion, the production of antimicrobial molecules, and the modulation of T cell responses.

## 1. Introduction

Recent studies have shown activated immune cells to undergo distinct metabolic changes, which not only fulfill the energy needs of these cells but also contribute to their effector function (reviewed in [1]). These metabolic changes are part of trained immune responses, which summarize the long-term functional reprogramming of innate immune cells evoked by exogenous or endogenous insults, leading to an altered response towards a second challenge after the return to a nonactivated state [2]. Innate immune cells undergoing trained immune responses can either mount stronger (trained immunity) or weaker (trained tolerance) immune responses mediated by a combination of epigenetic modifications and metabolic reprogramming of innate effector cells, as well as the long-term reprogramming of their bone marrow progenitor cells [2].

Accordingly, better understanding the mechanisms underlying trained immune responses will allow for a new generation of therapeutics and vaccines that combine trained immune responses in innate immune cells with the induction of classical antigen-specific adaptive immune responses.

As a model for such novel therapeutics in the field of allergies, we generated a recombinant fusion protein, rFlaA:Betv1, that combines the TLR5-ligand flagellin A from *Listeria monocytogenes* with the major birch pollen allergen Bet v 1 into a single molecule [3]. In previous studies, we were able to show that rFlaA:Betv1 efficiently suppressed allergic sensitization in vivo [3] while also activating myeloid DCs (mDCs) [3,4], macrophages [5], epithelial cells [6], and B cells [7], leading to pro-inflammatory cytokine production and the subsequent formation of allergen-specific TH1 responses with TH2-suppressing capacity [3,5]. Furthermore, rFlaA:Betv1 induced a prominent production of the anti-inflammatory cytokine IL-10 in all investigated types of antigen presenting cells (APCs) (mDCs, macrophages, and B cells), which was shown to significantly suppress TH2 responses [3,5,7].

Interestingly, previous research also showed both rFlaA:Betv1-stimulated mDCs and macrophages to undergo a metabolic switch towards a strongly glycolytic phenotype, resulting in lactate-induced acidification of the culture medium and a prominent color change in the contained pH indicator [3,5], referred to as the “Warburg Effect” [8]. Further analyses showed the anti-inflammatory IL-10 secretion by rFlaA:Betv1 to be dependent on the mechanistic target of rapamycin (mTOR), a master regulator of both cellular metabolism and immune function [3]. In contrast with this, rFlaA:Betv1-induced pro-inflammatory cytokine secretion in mDCs was shown to be largely mTOR independent, but depended on MAP kinase signaling [4]. In line with this, a differential role of metabolic pathways in the pro- and anti-inflammatory cytokine secretion induced by rFlaA:Betv1 has been proposed [3].

Although the metabolic changes that occur in immune cells activated by different stimuli are well described [9], both the activation of immune metabolism by potential future therapeutics, such as the fusion protein rFlaA:Betv1, and its contribution to the immune-modulating properties (such as cytokine secretion, influence on activation status, and antimicrobial properties) are not fully understood. Therefore, the aim of this study was to investigate the contribution of distinct metabolic pathways, including glycolysis, mitochondrial respiration, amino acid-, or fatty acid metabolism, to the rFlaA:Betv1-mediated activation of mDCs.

## 2. Results

### 2.1. TLR-Ligands Increase Glycolysis While Suppressing Oxidative Phosphorylation in mDCs

TLR-ligands were shown to robustly activate innate immune cells. To better understand how the activation of immune metabolism in mDCs by rFlaA:Betv1 compares to other well-known TLR-ligands, we stimulated mDCs with either Pam_2_CGDPKHPKSF (FSL-1) (TLR2/6-ligand), polyI:C (TLR3-ligand), Lipopolysaccharide (LPS) or monophosphoryl Lipid A (MPLA) (both TLR4-ligands), recombinant rFlaA (TLR5-ligand), the recombinant fusion protein rFlaA:Betv1 (TLR5-ligand), or R848 (TLR7- and TLR8-ligand) and analyzed both their metabolic phenotype and cytokine production (Repos. Figure S1A).

Metabolic activation of mDCs stimulated with the different TLR-ligands was investigated by determining the extracellular acidification rates (ECAR) as a proxy for increased glycolysis and oxygen consumption rates (OCR) as a marker for mitochondrial respiration (Repos. Figure S1A). With the exception of polyI:C, all investigated TLR-ligands strongly increased ECAR while simultaneously reducing OCR to varying degrees (Repos. Figure S1B). Notably, rFlaA:Betv1 induced both the strongest increase in ECAR- and decrease in OCR-values (Repos. Figure S1B, glycolytic vs. mitochondrial ATP production rates and overall contribution of glycolysis and oxidative phosphorylation to cellular metabolism determined in Repos. Figure S2). In line with the observed increases in extracellular acidification, stimulation of mDCs with either FSL-1, LPS, MPLA, rFlaA:Betv1, or R848 increased glycolytic ATP production rates and, therefore, the contribution of glycolysis to overall cellular metabolism (Repos. Figure S2).

In accordance with these results, all TLR-ligands (with the exception of polyI:C and rFlaA) induced a detectable Warburg Effect 72 h post-stimulation and increased glucose consumption from the culture medium (Repos. Figure S1C).

When analyzing cytokine secretion from stimulated mDCs, FSL-1, LPS, MPLA, rFlaA:Betv1, and R848 induced a robust cytokine response, while again, in the tested concentrations, polyI:C and rFlaA did not induce detectable secretion of the investigated cytokines (Repos. Figure S1D). Levels of cytokine secretion differed regarding the investigated TLR-ligands, with LPS and R848 consistently inducing the highest levels (IL-10: LPS: approx. 400 pg/mL (72 h post-stimulation); R848: approx. 500 pg/mL (72 h), IL-12p70: LPS: approx. 2400 pg/mL (24 h); R848: approx. 1600 pg/mL (24 h), IL-1β: LPS: approx. 2200 pg/mL (72 h); R848: approx. 500 pg/mL (72 h)), while FSL-1, MPLA, and rFlaA:Betv1 induced medium levels of the respective cytokines (IL-10: FSL-1: approx. 200 pg/mL (72 h); MPLA: approx. 35 pg/mL (72 h), rFlaA:Betv1: approx. 70 pg/mL (72 h), IL-12p70: FSL-1: approx. 170 pg/mL (24 h), MPLA: approx. 200 pg/mL (24h); rFlaA:Betv1: approx. 700 pg/mL (24 h), IL-1β: FSL-1: approx. 350 pg/mL (72 h), MPLA: approx. 500 pg/mL (72 h); rFlaA:Betv1: approx. 350 pg/mL (72 h)) (Repos. Figure S1D). IL-10 secretion was strongly increased from FSL-1-stimulated mDCs over time (10-fold increase comparing 24 h to 72 h), while for the other TLR-ligands the changes did not exceed more than a two-fold difference between both time points (Repos. Figure S1D). In contrast, IL-12p70 secretion declined over time, with all TLR-ligands showing lower levels of IL-12p70 72 h compared to 24 h post-stimulation (Repos. Figure S1D). Finally, IL-1β levels were consistently higher 72 h post-stimulation compared to 24 h post-stimulation (Repos. Figure S1D).

Collectively, these results show, that both the activation of mDC metabolism and effector function (in terms of cytokine secretion) is comparable between rFlaA:Betv1 and other well-established TLR-ligands. Therefore, rFlaA:Betv1 can be used as a model to study the immune–metabolic effects of certain TLR-ligand-containing fusion proteins as novel therapeutics.

### 2.2. rFlaA:Betv1 Induces a Pronounced Switch towards Glycolysis in Stimulated mDCs

**Figure 1:**
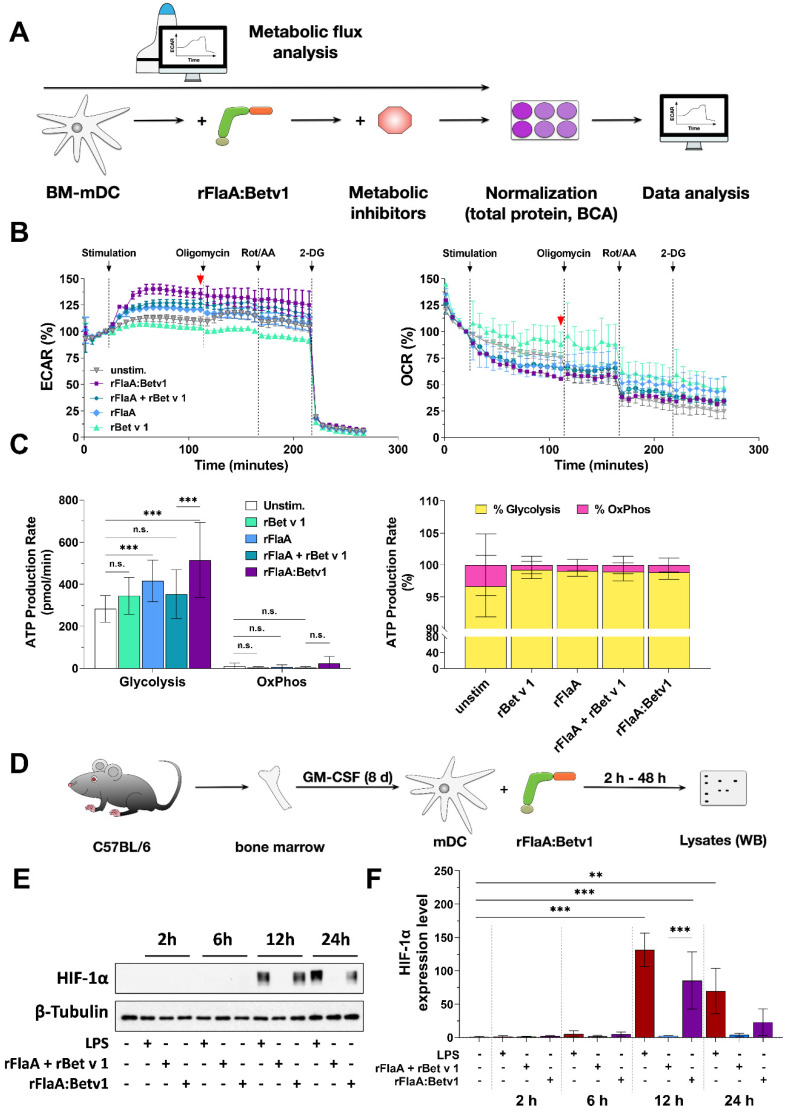
rFlaA:Betv1 induces a pronounced switch towards glycolysis in stimulated mDCs. C57Bl/6J mDCs were stimulated with equimolar concentrations of either rFlaA + rBet v 1 or rFlaA:Betv1 (equimolar to 4 µg/mL of rBet v 1) or 100 ng/mL LPS as a positive control and analyzed for extracellular acidification rates (ECAR) and oxygen consumption rates (OCR) using Seahorse technology (**A**). 14 cycles (84 min) post-stimulation, ATP synthase, the electron transport chain, and glycolysis were inhibited by sequential injection of oligomycin, rotenone/antimycin A (Rot/AA), and 2-deoxy-glucose (2-DG), for eight cycles (48 min) each, respectively. The red arrow indicates the time point used for statistical analyses in Repos. Figure S3 (for more information, see text) (**B**). Levels of glycolytic- and mitochondrial ATP-production, glycolysis, and oxidative phosphorylation (OxPhos) were analyzed by metabolic flux assay using the “ATP rate assay” according to the manufacturer’s recommendations (**C**). For HIF-1α detection, mDCs were stimulated with either 10 µg/mL of LPS, 17.4 µg/mL of rFlaA + 10 µg/mL rBet v 1, or 27.4 µg/mL rFlaA:Betv1 for the indicated time points and analyzed via Western Blot (**D**,**E**). Expression levels were first normalized to the β-tubulin loading control, followed by normalization to unstimulated controls (**F**). Data are either representative results of three independent experiments that showed similar results (**B**,**C**,**E**) or results of three independent experiments (**F**). Statistical comparisons were performed by 2-way ANOVA with correction for multiple comparisons according to Turkey and indicated as: n.s. *p*-value > 0.05, ** *p*-value < 0.01, *** *p*-value < 0.001.

To further characterize the metabolic phenotype of rFlaA:Betv1-stimulated mDCs, cells were stimulated with either the fusion protein or the equimolar mixture of both single proteins and analyzed for their metabolic phenotype by extracellular flux assay (Figure 1A). Here, rFlaA:Betv1 induced a significant increase in ECAR of approx. 40%, paralleled by a decrease in OCR of about 25% compared to unstimulated cells (Figure 1B, the time point indicated by a red arrow was quantified for statistical comparison in Repos. Figure S3B). These effects were less pronounced for the equimolar mixture of both single proteins (Figure 1B, Repos. Figure S3B).

To further elucidate the contribution of glycolysis and mitochondrial respiration to the observed effects, samples were consecutively injected with the ATP synthase inhibitor oligomycin, the complex I/III inhibitors rotenone and antimycin A (Rot/AA), and the hexokinase-2 inhibitor 2-deoxyglucose (2-DG) (Figure 1B). Inhibition of mitochondrial respiration resulted in the expected decrease in OCR in rFlaA + rBet v 1- and rFlaA:Betv1-stimulated samples (Figure 1B). Additional inhibition of glycolysis by 2-DG had no influence on OCR, but completely suppressed ECAR (Figure 1B). In line with these results, mDCs were found to be mainly glycolytic, with rFlaA:Betv1-stimulated mDCs displaying the highest glycolytic ATP production rates, while all tested stimulations decreased mitochondrial ATP production rates (Figure 1C).

To further exclude a contribution of mitochondrial respiration to rFlaA:Betv1-mediated mDC activation, rFlaA:Betv1-stimulated mDCs were pretreated with either oligomycin or antimycin A alone and checked for metabolic activation and cytokine secretion (Repos. Figure S4). Here, oligomycin had no influence on either rFlaA:Betv1-induced increase in ECAR, or decrease in OCR, while antimycin A induced a slight shift from OCR to ECAR (Repos. Figure S4A). Interestingly, inhibition of ATP synthase by oligomycin only increased IL-1β secretion, while antimycin A pretreatment only had a significant effect on IL-12p70 secretion, but not the other investigated cytokines (Repos. Figure S4A).

In order to characterize the intracellular signaling events associated with the observed changes in mDC activation and metabolic state, cells were stimulated with rFlaA:Betv1 and respective controls for either 2, 6, 12, or 24 h and analyzed by Western Blot (Figure 1D). In line with the observed switch to mainly glycolytic metabolism, we observed a distinct activation of hypoxia-inducible factor 1α (HIF-1α) in both LPS- and rFlaA:Betv1-stimulated but neither unstimulated nor rFlaA + rBet v 1-stimulated mDCs (Figure 1E,F).

In summary, rFlaA:Betv1-induced a pronounced increase in glycolytic metabolism which was independent of mitochondrial respiration.

### 2.3. rFlaA:Betv1-Induced mDC Activation Is Differentially Regulated by the Investigated Metabolic Pathways

**Figure 2:**
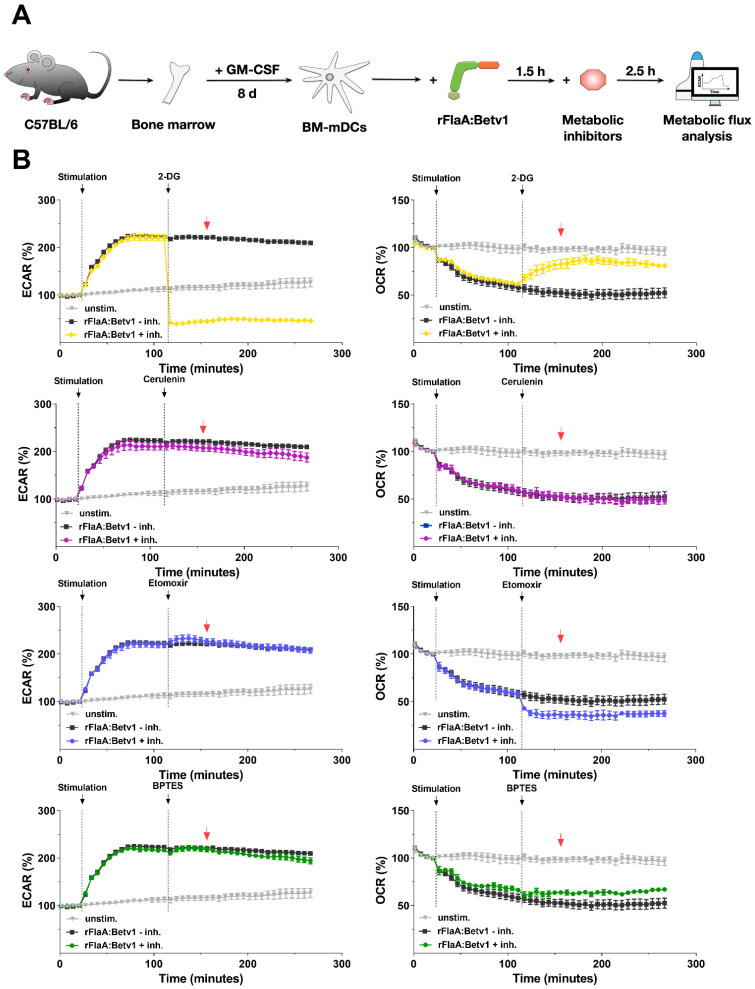
The rFlaA:Betv1-induced increase in ECAR depends on glucose metabolism. mDCs were stimulated with 11 µg/mL rFlaA:Betv1 for 14 cycles (84 min) followed by injection of the different inhibitors (2-DG: 50 mM, cerulenin: 2 µg/mL, etomoxir: 100 µM, BPTES: 20 µM) for another 24 cycles (144 min) and continuously analyzed for extracellular acidification rates (ECAR) and oxygen consumption rates (OCR) using Seahorse technology (**A**). Data are representative results of three independent experiments that showed similar results, with three to four technical replicates per experiment (**B**). The red arrow indicates the time point chosen for statistical analysis shown in Repos. Figure S5 (for more information, see text).

**Figure 3:**
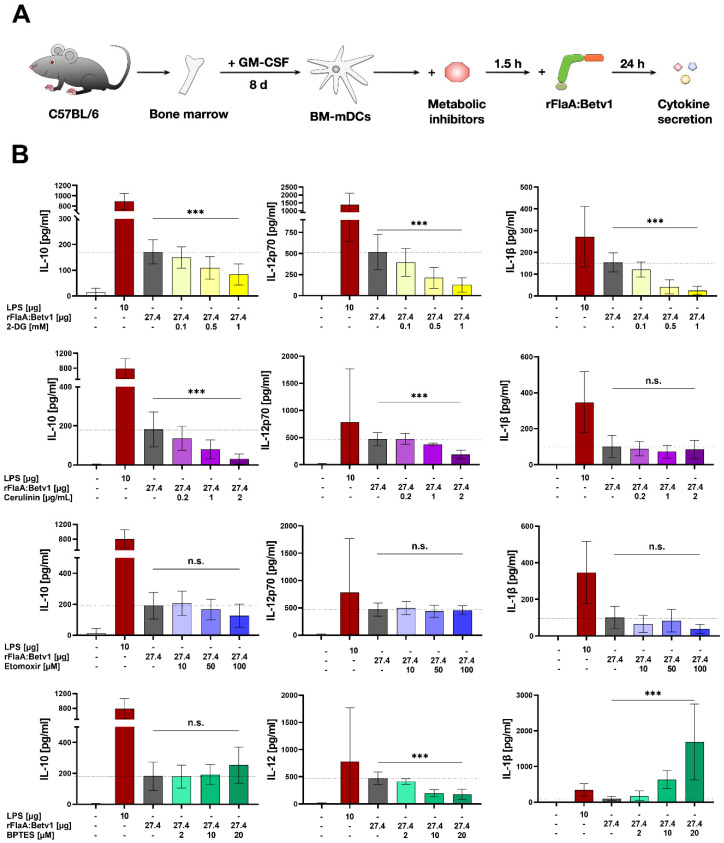
rFlaA:Betv1-induced IL-10 secretion from mDCs depends on glycolytic metabolism and fatty acid synthesis. mDCs were pretreated for 90 min with the indicated inhibitor concentrations followed by stimulation with 27.4 µg/mL of rFlaA:Betv1 for 24 h (**A**). Supernatants were analyzed for cytokine secretion by ELISA. Data are mean results of three to four independent experiments (**B**). Statistical comparisons were performed by 2-way ANOVA with correction for multiple comparisons according to Tukey and indicated as: n.s. *p*-value > 0.05, or *** *p*-value < 0.001. Please note that data shown in Figure 4 and Figure 5 were generated using the same mDC preparations.

**Figure 4:**
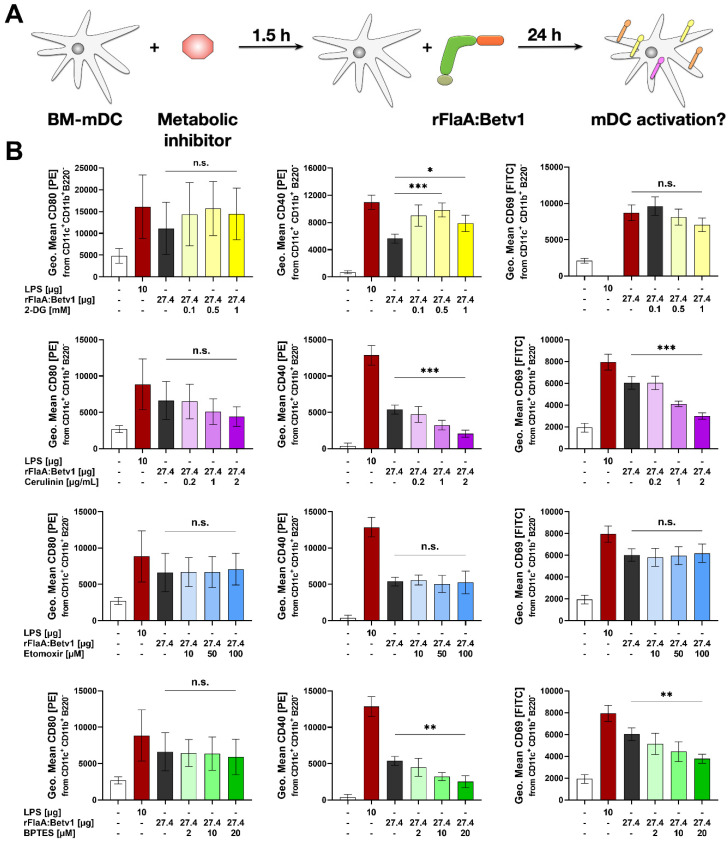
Glycolysis, fatty acid synthesis, and amino acid metabolism contribute to rFlaA:Betv1-induced expression of co-stimulatory molecules. mDCs were pretreated for 90 min with the indicated inhibitor concentrations, followed by stimulation with 27.4 µg/mL of rFlaA:Betv1 for 24 h (**A**). Cells were harvested, and surface expression of the indicated co-stimulatory molecules was quantified as geometric mean fluorescence intensities (MFI) by flow cytometry. Data are results of three to four independent experiments (**B**). Statistical comparisons were performed by 2-way ANOVA with correction for multiple comparisons according to Turkey and indicated as: n.s. *p*-value > 0.05, * *p*-value < 0.05, ** *p*-value < 0.01, or *** *p*-value < 0.001.

To explore the role of glycolysis, fatty acid-, and amino acid metabolism to rFlaA:Betv1-induced mDC activation, mDCs were treated with specific inhibitors and analyzed for the contribution of the investigated pathways to metabolic activation (Figure 2), cytokine secretion (Figure 3), and surface expression levels of activation markers (Figure 4). Please note that data shown in Figure 3 and Figure 4 were generated using the same mDC preparations.

To block glycolysis [10], 2-DG was used. Inhibition of fatty acid metabolism was achieved through pretreatment with either cerulenin to block fatty acid synthase [11] or etomoxir to block fatty acid oxidation by inhibiting carnitine palmitoyltransferase 1 [12]. Finally, for inhibition of amino acid metabolism, the glutaminase inhibitor bis-2-(5-phenylacetamido-1,3,4-thiadiazol-2-yl) ethyl sulfide (BPTES), which blocks the conversion of glutamine to glutamate within the mitochondrion, was employed [13].

To investigate the impact of the different metabolic pathways on the metabolic phenotype of mDCs via metabolic flux analyses, cells were first stimulated with rFlaA:Betv1 for 14 cycles to allow for the switch towards glycolysis, and then treated with the different inhibitors (Figure 2A). Here, 2-DG significantly suppressed the increase in ECAR, while at the same time inducing a compensatory increase in OCR (Figure 2B, the time point indicated by a red arrow is quantified for statistical comparison in Repos. Figure S5). Inhibition of fatty acid synthesis by cerulenin slightly, but not significantly, reduced glycolysis while having no effect on OCR (Figure 2B, Repos. Figure S5). In contrast, inhibition of fatty acid oxidation by etomoxir had no effect on the increase in ECAR, but further reduced OCR in rFlaA:Betv1-stimulated mDCs (Figure 2B, Repos. Figure S5). Finally, inhibition of glutaminase by BPTES significantly shifted the rFlaA:Betv1-induced increase in ECAR to an increase in OCR (Figure 2B, Repos. Figure S5).

mDCs were further investigated for their cytokine secretion pattern upon inhibition of the different metabolic pathways followed by rFlaA:Betv1-stimulation (Figure 3A). Treatment with 2-DG dose-dependently suppressed the secretion of all investigated cytokines 20 h post-stimulation (Figure 3B). Cerulenin pretreatment dose-dependently suppressed the rFlaA:Betv1-induced IL-10- and IL-12p70 secretion, while IL-1β secretion remained unchanged (Figure 3B). In contrast, etomoxir pretreatment had no effect on rFlaA:Betv1-induced cytokine secretion (Figure 3B). Notably, rFlaA:Betv1-induced IL-10 secretion was independent of amino acid metabolism, while inhibition by BPTES dose-dependently reduced IL-12p70 and strongly increased IL-1β secretion (Figure 3B).

Finally, the contribution of different metabolic pathways to the rFlaA:Betv1-induced increases in surface expression of CD80, CD40, and CD69 was investigated using CD11b^+^CD11c^+^B220^−^ mDCs (Figure 4A, gating strategy shown in Repos. Figure S6). Here, preincubation with 2-DG slightly, but not significantly, increased expression levels of CD80, while significantly enhancing CD40 expression. At the same time, no effect was observed for CD69 (quantified in Figure 4B; exemplary result shown in Repos. Figure S7). Cerulenin slightly reduced the expression levels of CD40 and CD69 by approx. 60% and 50%, respectively, while etomoxir had no effect on the expression levels of the investigated surface markers (Figure 4B, Repos. Figure S7). BPTES also reduced the expression levels of CD40 to approx. 45% and CD69 to 60% compared to the original expression levels observed in rFlaA:Betv1-stimulated mDCs (Figure 4, Repos. Figure S7).

In summary, the in-depth analysis of metabolic pathways contributing to rFlaA:Betv1-mediated activation of mDCs showed a strong dependency of both metabolism and cytokine secretion on glycolysis. Interestingly, expression of all co-stimulatory molecules investigated, IL-10-, and IL-12 secretion depended on fatty acid synthesis, while IL-1β secretion, CD40-, and CD69-expression were shown to be connected to amino acid metabolism.

### 2.4. rFlaA:Betv1 Triggers a mTOR- and Fatty Acid Metabolism-Dependent Secretion of Antimicrobial Factors from Stimulated mDCs

A predominant switch to glycolysis induced by different activating stimuli was shown to be paralleled by reduced mitochondrial activity and a “disrupted” Krebs cycle [14]. In activated immune cells, this “disrupted” Krebs cycle is used to produce different secondary metabolites with both direct and indirect antimicrobial activity, such as reactive oxygen species (ROS), NOS, prostaglandins, or itaconate [15].

**Figure 5:**
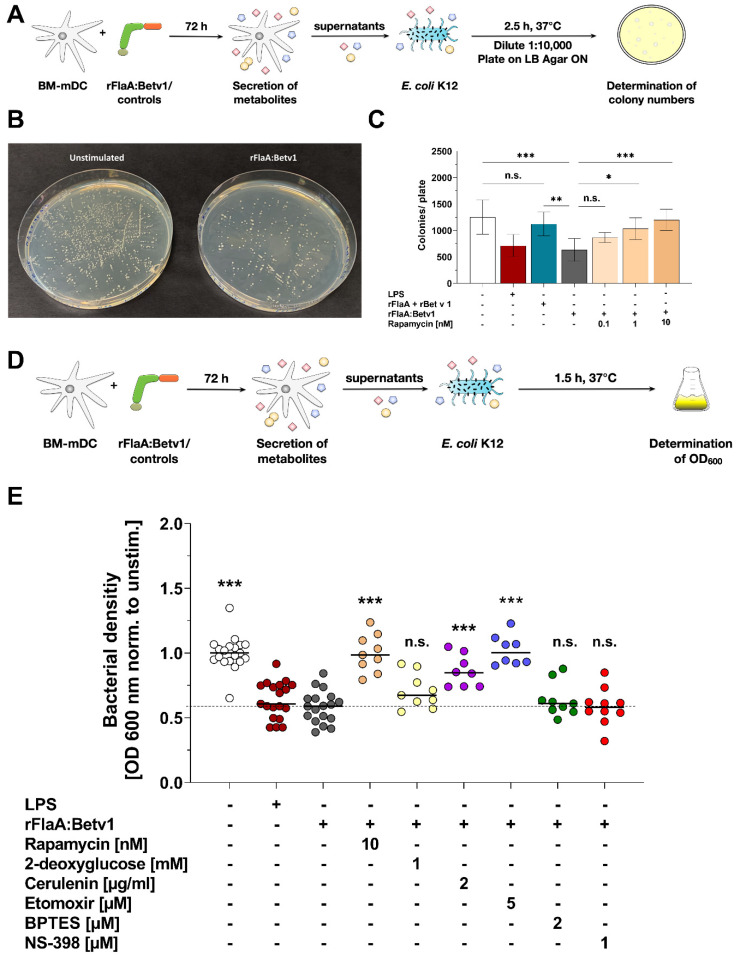
rFlaA:Betv1 triggers a mTOR- and fatty acid metabolism-dependent secretion of antimicrobial factors from stimulated mDCs. *E. coli* K12 cultures were incubated for 2.5 h with supernatants derived from mDCs stimulated as indicated and plated overnight on agar plates (**A**). Bacterial growth was documented photographically (**B**) and colony numbers were counted manually (**C**). Bacterial growth in liquid cultures was determined photometrically by measuring the OD_600nm_ 1.5 h post-addition of the indicated supernatants (**D**,**E**). Data are either representative results of four independent experiments that showed similar results (**B**) or results of three to four independent experiments (**C**,**E**). Statistical comparisons were performed by 2-way ANOVA with correction for multiple comparisons according to Turkey and indicated as: n.s. *p*-value > 0.05, * *p*-value < 0.05, ** *p*-value < 0.01, or *** *p*-value < 0.001.

To test whether the investigated switch towards glycolysis and the reduced mitochondrial activity in rFlaA:Betv1-stimulated mDCs (Figure 1) results in the secretion of antimicrobial factors, we studied the effects of supernatants derived from rFlaA:Betv1-stimulated mDCs on *E. coli* growth (Figure 5). For this purpose, *E. coli* K12 cultures were incubated for 2.5 h with supernatants of mDCs that were previously stimulated for 72 h with either rFlaA:Betv1 or the respective controls. Subsequently, bacteria were plated on agar plates, and the number of colonies was determined the next day (Figure 5A). Supernatant derived from rFlaA:Betv1-stimulated mDCs suppressed bacterial growth (exemplary plates shown in Figure 5B and quantified in C), while pretreatment of mDCs with the mTOR-inhibitor rapamycin prior to stimulation with rFlaA:Betv1 dose-dependently restored bacterial growth (Figure 5C).

Furthermore, we quantified bacterial growth in liquid cultures based on OD_600_ measurements for easier handling (Figure 5D). Supernatants of mDCs, which were previously treated with either the fusion protein rFlaA:Betv1 or LPS, suppressed bacterial growth compared to supernatants derived from unstimulated mDCs (Figure 5E). This effect was abolished when the cells were either pretreated with the mTOR-inhibitor rapamycin or the inhibitors of fatty acid metabolism cerulenin or etomoxir (Figure 5E). Here, no significant changes in antimicrobial activity were observed upon application of supernatants derived from mDCs that were pretreated with either hexokinase 2-inhibitor 2-DG, glutaminase inhibitor BPTES, or COX2-inhibitor NS-398, which is involved in the conversion of arachidonic acid to prostaglandins (Figure 5E).

In summary, these results show the metabolic changes in rFlaA:Betv1-stimulated mDCs to promote the production of secreted factors with direct antimicrobial activity. Among the tested metabolic pathways, mTOR and fatty acid metabolism were shown to be especially important for the production of these antimicrobial substances.

### 2.5. Fatty Acid Synthesis Contributes to the T Cell Modulating Properties of rFlaA:Betv1-Stimulated mDCs

**Figure 6:**
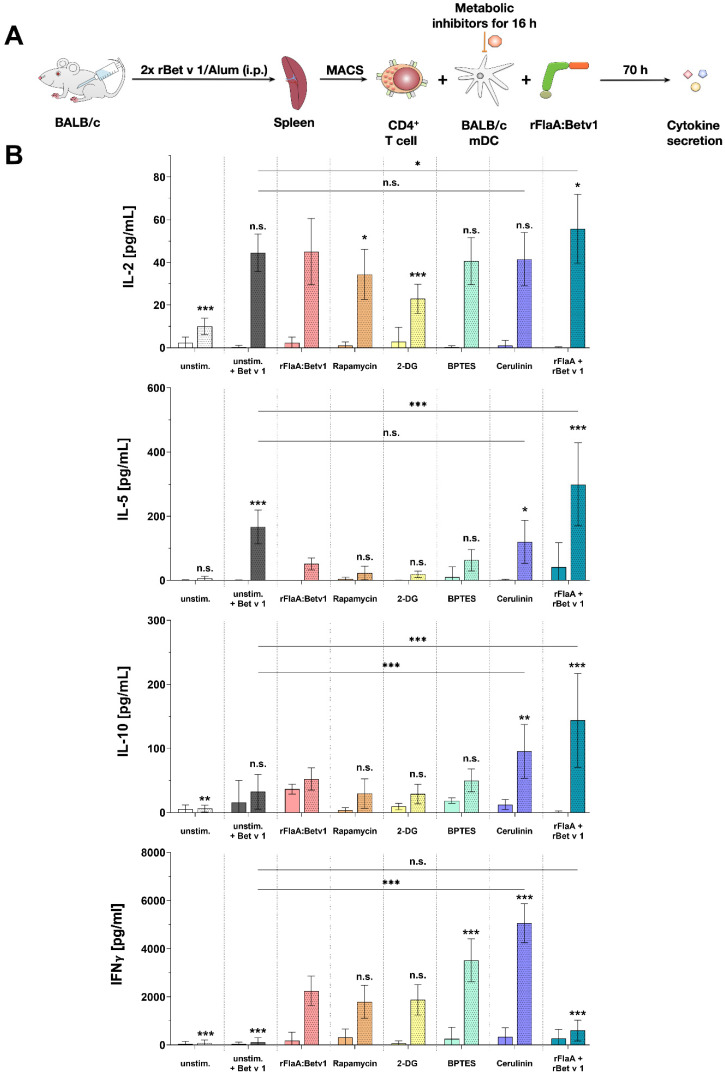
Fatty acid metabolism contributes to the T cell modulating properties of rFlaA:Betv1-stimulated mDCs. BALB/c mDCs were co-cultured with CD4^+^ T cells isolated from spleens of BALB/c mice that were previously immunized with the major birch pollen allergen Bet v 1 and alum. Prior to co-cultures, mDCs were pretreated for 16 h with metabolic inhibitors (5 nM rapamycin, 0.5 mM 2-DG, 1 µM BPTES, or 0.2 µg/mL cerulenin), then the medium was changed. mDCs were either left alone (solid bars) or T cells were added (bars with dotted pattern). Cultures were either left unstimulated or re-stimulated with either 2 µg/mL Bet v 1 with and without the addition of equimolar amounts of either rFlaA:Betv1 or rFlaA+Betv1 for additional 70 h (**A**). Cytokine secretion was determined for IL-2, IL-10, IL-5, and IFN-γ (**B**). Data are mean results of four independent experiments±SD. Statistical comparisons were performed by 2-way ANOVA with correction for multiple comparisons according to Dunnett and indicated as: n.s. *p*-value > 0.05, * *p*-value < 0.05, ** *p*-value < 0.01, or *** *p*-value < 0.001.

To determine the contribution of the rFlaA:Betv1-mediated metabolic changes to their T cell priming capacity, mDCs were differentiated from BALB/c bone marrow and co-cultured with T cells that were isolated from spleens of BALB/c mice immunized against the major birch pollen allergen Bet v 1 using alum (Figure 6A).

mDCs which were not co-cultured with T cells showed no secretion of either IL-2, IL-5, or IFN-γ, while re-stimulation of the co-cultures with Bet v 1 triggered the secretion of IL-2 and IL-5, but also the anti-inflammatory cytokine IL-10 (Figure 6B). As shown before, mDCs stimulated with rFlaA:Betv1 secreted IL-10 (Figure 6B). Addition of rFlaA:Betv1 to Bet v 1-restimulated co-cultures suppressed the Bet v 1-induced IL-5 secretion by approx. 65% (Figure 6B).

Pretreatment of the mDCs with either the mTOR-inhibitor rapamycin or the hexokinase 2-inhibitor 2-DG efficiently suppressed mDC-derived IL-10 secretion and resulted in slightly suppressed Bet v 1 + rFlaA:Betv1-induced IL-2 secretion from co-cultures (Figure 6B). rFlaA:Betv1-induced IFN-γ secretion was not influenced by either rapamycin or 2-DG (Figure 6B). Inhibition of amino acid metabolism by pretreatment of mDCs with BPTES significantly increased rFlaA:Betv1-induced IFN-γ secretion in co-cultures (Figure 6B). Inhibition of fatty acid synthesis by cerulenin strongly enhanced Bet v 1 + rFlaA:Betv1 induced IL-10 and IFN-γ secretion, while reversing the suppression of IL-5 secretion (Figure 6B). In contrast with the fusion protein, the mixture of both single proteins significantly boosted IL-2, IL-5, and IL-10, but not IFN-γ secretion in co-cultures compared to cells only stimulated with Bet v 1 (Figure 6B).

## 3. Discussion

Over the last few years, the induction and modulation of trained immune responses in innate immune cells has become a topic of great interest in the vaccination field. Trained immunity describes the heightened reactivity of innate immune cells after prior activation either to the same or an unrelated stimulus [2]. It is based on metabolic and epigenetic changes in both terminally differentiated immune cells, as well as their bone marrow progenitors [2]. In addition to whole pathogens, TLR-ligands and C-type lectins were among the first described stimuli to trigger trained immune responses, making them interesting adjuvant candidates [16,17].

We recently generated and characterized a fusion protein consisting of the TLR5-ligand flagellin and the major birch pollen allergen Bet v 1 (rFlaA:Betv1) [3], which was meanwhile shown to suppress TH2 responses in vitro and allergic sensitization in vivo [3]. These immune modulating properties of rFlaA:Betv1 were paralleled by a pronounced activation of glycolytic metabolism in both mDCs [3] and macrophages [5], as well as a strong activation of epithelial cells [6] and B cells [7].

In the present study, we compared the immune–metabolic changes induced by rFlaA:Betv1 in mDCs to other well-established TLR-ligands. In addition to nonreactivity of the investigated mDCs to polyI:C, likely caused by these cells expressing only low levels of the corresponding pattern recognition receptor TLR3 [18], all other TLR-ligands induced a shift towards glycolytic metabolism (indicated by enhanced ECAR values) while also reducing oxygen consumption (indicated by decreases in OCR) to varying degrees. The same metabolic response was observed for rFlaA:Betv1-stimulated mDCs, making rFlaA:Betv1 a suitable model to study the immune metabolic effects of novel vaccine candidates that incorporate antigens.

We then further characterized the metabolic phenotype of rFlaA:Betv1-stimulated mDCs in more detail. The increased glycolysis in rFlaA:Betv1-stimulated mDCs was paralleled by a marked decrease in mitochondrial respiration. Furthermore, while inhibition of glycolysis abrogated the observed metabolic switch and dose-dependently suppressed secretion of all investigated cytokines, inhibition of the mitochondrion by antimycin A further enhanced the rFlaA:Betv1-induced metabolic switch towards glycolysis. Here, neither the inhibition of ATP synthase by oligomycin nor inhibition of complex III with antimycin A had an effect on the secretion of the anti-inflammatory cytokine IL-10.

Biochemically, the shift towards preferred lactate production from pyruvate is mediated via a HIF-1α-dependent upregulation of different pyruvate dehydrogenase kinases (PDHK) [19]. In addition, the upregulation of glycolytic enzymes via HIF-1α activation further increases the speed of glycolysis [20]. Indeed, Western Blot analyses of rFlaA:Betv1-stimulated mDCs showed a distinct upregulation of HIF-1α both 12 and 24 h post-stimulation.

Under hypoxic conditions, HIF-1α promotes the expression of many genes, but is rapidly degraded by the ubiquitin proteasome pathway under ambient conditions due to its oxygen sensitivity [21]. In addition, immune cell activation under normoxic conditions can also result in HIF-1α stabilization. For example, Tannahill et al. first reported that LPS-stimulation of macrophages led to the accumulation of the Krebs cycle intermediate succinate via glutamine-dependent anaplerosis and the “GABA-shunt” pathway [22]. In this context, succinate acted as a cell-intrinsic danger signal, driving both glycolysis and pro-inflammatory IL-1β production via stabilization of HIF-1α (termed “pseudohypoxia”) [22,23]. Interestingly, the inhibition of glycolysis using 2-DG decreased the amount of rFlaA:Betv1-induced IL-10 and IL-1β. In contrast, inhibition of HIF-1α activity by either blocking HIF-1α expression using YC-I or enhancing the degradation of HIF-1α by DBA had no impact on the production of IL-1β. At the same time, both inhibitors led to increased secretion of IL-10 (Repos. Figure S8B). In this experimental setting, only YC-I decreased rFlaA:Betv1-induced IL-12p70 secretion (Repos. Figure S8B). None of the used HIF-1α-inhibitors significantly affected rFlaA:Betv1-induced antimicrobial activity (Repos. Figure S8C). These results suggest that, while levels of IL-10 and IL-12p70 are connected to HIF-1α signaling in rFlaA:Betv1-stimulated mDCs, both IL-1β production and antimicrobial activity are independent of HIF-1α and may be triggered by different mechanisms such as mitochondrial stress and the assembly of the inflammasome (see below).

Moreover, inhibition of glutaminase as part of amino acid metabolism by BPTES slightly reduced the rFlaA:Betv1-induced increase in ECAR and slightly increased OCR. rFlaA:Betv1-induced IL-10 secretion was independent of BPTES pretreatment while IL-12p70 was dose-dependently reduced, and IL-1β secretion strongly increased by BPTES. Besides, BPTES also reduced the expression levels of CD40 and CD69.

Mechanistically, BPTES blocks glutaminase (GLS1), which converts glutamine to glutamate and ammonia, leading to an increase in cellular glutamine levels [24]. As well as being imported into the mitochondrion, glutamine can also be used for the generation of uridine diphosphate-N-acetyl-glucosamine in the cytoplasm, which is an essential substrate for subsequent glycosylation reactions, making the availability of glutamine crucial to maintain proper protein function as well as signal transduction through glycosylation reactions [25,26].

In combination with mitochondrial ROS generated by mitochondrial damage, glutamine can further promote the assembly of the NLRP3 inflammasome complex [27], enhancing IL-1β production. This theory is supported by the fact that, in addition to BPTES, oligomycin pretreatment in rFlaA:Betv1-stimulated mDCs also increased IL-1β production, presumably caused by increased rates of mitochondrial oxidative stress.

In line with this complex network, BPTES was described to have pleiotropic effects affecting numerous metabolic pathways, including glycolysis, the Krebs cycle, as well as nucleotide and amino acid metabolism in two breast cancer cell lines [28], suggesting the effects of BPTES on overall cellular metabolism to be more complex than the simple inhibition of glutamate generation in the mitochondrion.

We further analyzed the contribution of fatty acid metabolism to the observed activation of mDCs by rFlaA:Betv1. Metabolically, only inhibition of fatty acid synthesis by cerulenin slightly reduced glycolysis (while having no effect on OCR). Moreover, cerulenin pretreatment dose-dependently suppressed the rFlaA:Betv1-induced IL-10- and IL-12p70 secretion, while IL-1β secretion remained unchanged. In contrast, etomoxir pretreatment slightly reduced mitochondrial oxygen consumption and interfered with antimicrobial activity otherwise observed in supernatants of rFlaA:Betv1-stimulated mDCs, but had no impact on the investigated ECAR, cytokine secretion, or expression of co-stimulatory molecules. Etomoxir concentrations greater than 5 µM were recently shown to induce mitochondrial stress associated with ROS production in human T cells independently of its effect on carnitine palmitoyltransferase 1 (CPT1a) [29]. On the basis of these results, similar off-target effects of etomoxir in mDCs cannot be excluded. Therefore, our results obtained in mDCs pre-treated with etomoxir have to be interpreted cautiously.

It is described that in activated immune cells, a metabolic switch towards glycolysis is usually paralleled by a “disrupted” mitochondrial Krebs cycle resulting from an insufficient supply of the mitochondrion with pyruvate (which is preferentially used to regenerate NAD via the generation of lactate). This “disrupted” Krebs cycle was shown to facilitate the generation of both directly and indirectly antimicrobial molecules such as prostaglandins, nitric oxide (NO), ROS, or itaconate, all of which are important immune effector molecules (reviewed in [15]).

Indeed, we observed a direct antimicrobial activity of supernatants derived from rFlaA:Betv1-stimulated mDCs on *E. coli* K12 cultures. This antimicrobial activity was shown to depend on both mTOR signaling and fatty acid metabolism as inhibition of either fatty acid oxidation, fatty acid synthesis, or mTOR was shown to prevent mDCs from producing these antimicrobial molecules.

Currently, the exact mechanisms of the observed antimicrobial activity are unclear. Recently, mTOR-dependent autophagy has emerged as an innate immune response pathway that contributes to antimicrobial activity by targeting cytosolic bacteria to autophagosomes in order to restrict bacterial growth (reviewed in [30]). Therefore, mTOR-dependent autophagy of the bacteria inside the rFlaA:Betv1-stimulated mDCs could contribute to antibacterial responses.

Additionally, as we still observed antimicrobial activity in cell-free, previously frozen supernatants of rFlaA:Betv1-stimulated mDCs and antibacterial activity was shown to be dependent on both mTOR and fatty acid metabolism, our results suggest that the production of antimicrobial molecules requires the mTOR-dependent switch in cellular metabolism which results in the above-described “disrupted” Krebs cycle. The intermediates accumulating from the “disrupted” Krebs cycle are the building blocks of important immune active and antimicrobial compounds [31].

One example is the accumulation of citrate, cis-aconitate, and D-isocitrate (caused by decreased expression of the enzyme isocitrate dehydrogenase within the “disrupted” Krebs cycle, which normally converts D-isocitrate to α-ketoglutarate), resulting in the generation of prostaglandins, NO, ROS, or itaconate (reviewed in [15]). In particular, itaconate, which is produced from citrate by activated DCs (and macrophages) has been reported not only to have antibacterial activity, but also to reduce inflammation by inhibiting succinate dehydrogenase [32]. Furthermore, a recent study by Jaiswal and colleagues was able to identify itaconate as a key Krebs cycle intermediate with the ability to reduce TH2 cytokine production [33]. Therefore, itaconate could play a role in the immune-modulating properties of rFlaA:Betv1-stimulated mDCs.

Moreover, the formation of certain antimicrobial metabolites such as prostaglandins depends on fatty acid metabolism. As a precursor, fatty acids are metabolized into either arachidonic acid or eicopentaenoic acid, which are subsequently turned into prostaglandins and thromboxanes via cyclooxygenases 1 and 2 (COX1/2) [34]. In an initial experiment, we analyzed if the antimicrobial effect of rFlaA:Betv1-stimulated mDC-derived supernatants was caused by the production of prostaglandins, by inhibiting COX2 using NS-398. Here, inhibition of COX2 by NS-398 did not modify the rFlaA:Betv1-induced antimicrobial effect (Figure 5E) and only slightly reduced IL-12p70 secretion (Repos. Figure S8B). As the bacterial density of cultures incubated with NS-398 pretreated rFlaA:Betv1-stimulated mDC supernatants was equal to those incubated with simply rFlaA:Betv1-stimulated mDC supernatants, we suggest that molecules other than prostaglandins generated from Krebs cycle intermediates, such as, e.g., itaconate, ROS, or NOS, could be responsible for the antimicrobial activity observed in rFlaA:Betv1-stimulated mDC supernatants. In addition, arachidonic acid and other unsaturated fatty acids can also directly function as endogenous antimicrobial molecules [35]. Therefore, the observed dependency of antimicrobial factor secretion on both mTOR activation and fatty acid metabolism is in line with the reported biochemical alterations occurring in predominantly glycolytic, activated innate immune cells. Nevertheless, we could show the stimulation of mDCs with rFlaA:Betv1 to result in the secretion of antimicrobial molecules, but did not identify the respective substances. Elucidating which molecules mediated the observed antimicrobial activity will be the focus of additional studies.

Finally, we investigated the contribution of the different mDC metabolic pathways to their capacity to modulate antigen-specific T cell responses. As reported before, the addition of rFlaA:Betv1 to rBet v 1-stimulated co-cultures was shown to suppress the Bet v 1-induced secretion of the TH2 cytokine IL-5 while maintaining IL-2, IFN-γ, and IL-10 secretion [3]. Interestingly, only inhibition of fatty acid metabolism by cerulenin pretreatment of rFlaA:Betv1-stimulated mDCs reversed the suppression of IL-5 production while increasing secretion levels of both IL-10 and IFN-γ. Furthermore, IFN-γ secretion in co-cultures was also increased by inhibition of either mTOR, glucose, or amino acid metabolism. Collectively, these results suggest that fatty acid metabolism and its derived metabolites in mDCs with a “disrupted” Krebs cycle are highly important for the suppression of TH2 responses.

One shortcoming of our study is that we did not analyze the CD4^+^ T cells used for the co-culture experiments in more detail. While we observed an induction of both TH1 and TH2-cytokine secretion after re-stimulation of the T cells ex vivo, showing that the sensitization induced Bet v 1-specific T cells, we cannot comment on the frequency of allergen-specific T cells. Furthermore, we did not analyze a potential induction of regulatory T cells by the rFlaA:Betv1-stimulated mDCs. These parameters will be the focus of future studies.

Although we have only examined acute mDC activation in our study, the strong activation of mDCs by the fusion protein in conjunction with the distinct changes in mDC metabolism (which were comparable to other well-established TLR-ligands already described to induce trained immune responses) allows us to speculate that rFlaA:Betv1 may be able to induce trained immune responses. Investigating if such trained immune responses are indeed induced and may mediate heterologous protection against other antigens upon in vivo application of flagellin-containing fusion proteins will be a focus of future studies.

## 4. Material and Methods

### 4.1. Mice

BALB/c and C57Bl/6 (Jackson Laboratories, Bar Harbor, ME, USA) were bred under specific pathogen-free (SPF) conditions at the animal facility of the Paul-Ehrlich-Institut. All animal experiments were performed according to the German animal protection law (granting authority: RP Darmstadt, Germany; approval no. F107/1049).

### 4.2. Generation of Recombinant Proteins

rFlaA from *L. monocytogenes* (accession no: NC_003210) was generated according to Schülke et al. [36], recombinant birch pollen allergen Bet v 1 (accession no: X15877.1) according to the method of Siebeneicher et al. [37], and the fusion protein of rFlaA and rBet v 1 (rFlaA:Betv1) was generated according to Schülke et al. [3] using cDNA fusion with cDNAs of both rFlaA and rBet v 1 as templates.

### 4.3. TLR-Ligands

LPS was obtained from Sigma-Aldrich (derived from *Salmonella abortus equi*, L5886, Steinheim, Germany). All other used TLR-ligands (FSL-1, poly I:C, MPLA, and R848) were obtained from InvivoGen (Toulouse, France). rFlaA and rFlaA:Betv1 were generated recombinantly (see above). TLR-ligands were used, if not stated otherwise, in the following concentrations: FSL-1 1 µg/mL; poly I:C 10 µg/mL, LPS 10 µg/mL; MPLA 10 µg/mL; rFlaA 17.4 µg/mL; rFlaA:Betv1 27.4 µg/mL; or R848 1 µg/mL. mDCs were stimulated with the different TLR-ligands for either 24 or 72 h as independent stimulations and analyzed for cytokine secretion.

### 4.4. Differentiation and In Vitro Stimulation of mDCs

Mouse mDCs were generated as described previously [36]. Bone marrow cells from either C57Bl/6 or BALB/c mice were cultured in culture medium (RPMI1640, Gibco, Karlsruhe, Germany, supplemented with 10% FCS, 1 mM sodium pyruvate, 10 mM HEPES, penicillin (100 U/mL), streptomycin (100 µg/mL), and 0.1 mM 2-mercaptoethanol, and 100 ng/mL recombinant mouse GM-CSF) for 8 days, and medium was changed every 2 days. On day 8, mDCs were harvested by pipetting and either analyzed by Metabolic Flux Analysis (details see below) or seeded at a concentration of 5 × 10^5^ cells/mL in 24-well plates (Thermo Scientific, Dreieich, Germany) in culture medium without the supplementation of GM-CSF.

### 4.5. ELISA Measurements

Cytokine concentrations in supernatants were analyzed by ELISA using the following antibody combinations: IL-2: purified anti-mouse IL-2 (clone: JES6–1A12, 1:500) and biotin anti-mouse IL-2 (JES6–5H4, 1:500, both BioLegend, San Diego, CA, USA); IL-10: mouse IL-10 ELISA Development Kit (#900-T53, PeproTech, Hamburg, Germany); IL-1β: purified anti-mouse IL-1β (clone: B122, 1:500) and biotin anti-mouse IL-1β (polyclonal, 1:500); IL-5: purified anti-mouse IL-5 (clone: TRFK5, 1:500) and biotin anti-mouse IL-5 (clone: TRFK4, 1:500); IL-12: purified anti-mouse IL-12p70 (clone: C18.2, 1:500) and biotin anti-mouse IL-12p70 (clone: C17.8, 1:500); IFN-γ: purified anti-mouse IFN-γ (clone: XMG1.2, 1:1000) and biotin anti-mouse IFN-γ (clone: R4-6A2, 1:500); IL-13: purified anti-mouse IL-13 (clone: eBio13A; 1:500) and biotin anti-mouse IL-13 (clone: eBio1316H, 1:250, all eBioscience, Frankfurt, Germany).

### 4.6. Flow Cytometry

rFlaA:Betv1-induced mDC activation was analyzed by flow cytometry using anti-mouse PE-conjugated CD40 (clone: 1C10, dilution: 1 to 100), PE-conjugated CD69 (clone: H1.2F3, dilution: 1 to 150), FITC-conjugated CD80 (clone: 16–10A1, dilution: 1 to 50, all eBiosciences, Frankfurt, Germany). Additionally, cells were stained with anti-mouse pacific blue-conjugated CD11b (clone: M1/70.15, dilution: 1 to 50, Invitrogen, Thermo Fisher Scientific), allophycocyanin-conjugated CD11c (clone: HL3, dilution: 1 to 500, BD Bioscience), and PE-Cy5-conjugated B220 (clone: RA3–6B2, dilution: 1 to 100, BD Bioscience). The FITC- or PE-intensity of CD11b^+^CD11c^+^B220^−^ cells (mDCs) was quantified by flow cytometry using a FORTESSA flow cytometer (BD Bioscience). Geometrical mean fluorescence intensities were calculated and normalized to unstimulated cells. Data were analyzed using FlowJo V.7 (Treestar Inc., Ashland, OR, USA).

### 4.7. Metabolic Inhibitor Experiments

If indicated, mDCs were pretreated for 90 min with either 0.1 or 1 µg/mL antimycin A from *Streptomyces* sp. (electron transport chain complex III inhibitor), 0.02, 0.2, or 2 µM oligomycin from *Streptomyces diastatochromogenes* (inhibitor of ATP synthase), 1 µM NS-398 (COX-2 inhibitor, involved in the conversion of arachidonic acid to prostaglandin H2), 0.1, 1, or 10 nM rapamycin (mTOR inhibitor), 2, 10, or 20 µM BPTES (glutaminase inhibitor), 0.2, 1, or 2 µg/mL cerulenin (inhibitor of fatty acid synthase), 10, 50, or 100 µM etomoxir (inhibitor of carnitine palmitoyltransferase 1 (CPT1a) involved in fatty acid oxidation, all Sigma-Aldrich, Steinheim, Germany), 0.1, 0.5, or 1 mM 2-deoxyglucose (2-DG, hexokinase-2 inhibitor, Carl-Roth Laboratory Supplies, Karlsruhe, Germany), 0.1, 1, or 10 µM YC-I (post-transcriptional inhibitor of HIF-1α expression), or 1, or 10 µM dimethyl-bisphenol A (DBA, HIF-1α inhibitor, promoting degradation of HIF-1α protein, both Abcam). Subsequently, mDCs were either left unstimulated or stimulated as indicated for either 24 h (to determine cytokine secretion by ELISA or expression of surface markers by flow cytometry) or 72 h (determination of cytokine secretion, Warburg Effect, glucose consumption, and antimicrobial activity). Stimulation was done with either 17.4 µg/mL rFlaA + 10 µg/mL rBet v 1, or 27.4 µg/mL rFlaA:Betv1. LPS (10 µg/mL, L5886, Sigma Aldrich, Taufkirchen, Germany) was used as a positive control. The toxicity of the used inhibitors was determined using the fixable viability dye eFlour780 (Thermo Fisher Scientific, data not shown). Inhibitor concentrations showing toxic effects were excluded from the analyses.

### 4.8. Western Blot

1.5 × 10^6^ mDCs were seeded and starved for 2 h in RPMI plus 1% of FBS in 6-well plates (Thermo Scientific), and then stimulated with either 10 µg/mL LPS, 17.4 µg/mL rFlaA + 10 µg/mL rBet v 1, or 27.4 µg/mL rFlaA:Betv1 for the indicated durations. Cells were harvested, lysed with lysis buffer (62.5 mM Tris-HCl (pH 6.8), 2% *w*/*v* SDS, 10% glycerol, 50 mM DTT, 0.01% *w*/*v* bromophenol blue), and proteins were separated by SDS-polyacrylamide gel followed by transfer to nitrocellulose membranes. The expression of hypoxia-inducible factor 1α (HIF-1α) was analyzed using: 1st Ab: anti-HIF-1α antibody (Cell Signaling Technologies (CST), Cat #14179S, 1:1000) and 2nd Ab: anti-rabbit IgG, HRP-conjugated antibody (CST, Cat #7074, 1:5000). β-tubulin was detected using: HRP-conjugated anti-β-tubulin (CST, Cat #5346, 1:5000). Images were captured with an iBright™ CL1500 system (Thermo Fischer Scientific), and the intensities of band were quantified using ImageJ software (imagej. nih.gov, version: 1.52a).

### 4.9. Generation of TH2-Biased rBet v 1-Specifc CD4^+^ T Cells

For the generation of Bet v 1-specific, TH2-biased CD4^+^ T cells, BALB/c mice were sensitized by two i.p. injections of 10 µg rBet v 1 plus 2 mg aluminum hydroxide (alum; Inject^TM^ Alum, Thermo Scientific, Dreieich, Germany) two weeks apart. One week after the second sensitization, mice were sacrificed, and splenocytes were used for CD4^+^ T cell isolation using the CD4 T Cell Isolation Kit according to the manufacturer’s recommendations (Miltenyi Biotec, Bergisch Gladbach, Germany).

### 4.10. mDC:TC Co-Cultures

First, 1.6 × 10^5^ BALB/c mDCs were seeded in 48-well plates (Thermo Scientific). mDCs were treated with different metabolic inhibitors (5 nM rapamycin, 1 µM BPTES, 0.5 mM 2-deoxyglucose, or 0.2 µg/mL cerulenin) for 16 h. Subsequently, plates were washed by centrifugation at 1200 rpm for 5 min at 4 °C, media were removed carefully through pipetting, and 250 µL of fresh RPMI culture media was added. T cells were isolated from rBet v 1/alum-sensitized BALB/c mice as described above and 6.3 × 10^5^ CD4^+^ T cells were added to the mDCs. Co-cultures were stimulated with either 10 µg/mL LPS or 13.7 µg/mL rFlaA:Betv1. Co-cultures were subsequently either left unstimulated or re-stimulated with 2 µg/mL of rBet v 1 for 72 h. The total culture volume was adjusted to 500 µL. Cytokine secretion in culture supernatants was determined by ELISA. Please note that unstimulated and rBet v 1-stimulated controls shown in Figure 6 were shared with a previously published dataset [38] for 3R reasons, while all other results contained in Figure 6 were previously unpublished.

### 4.11. Determination of Antimicrobial Activity

To determine the antimicrobial activity of supernatants derived from mDCs after 72 h of culture, overnight cultures (ONC) of *Escherichia coli* (*E. coli*) K12 were prepared. To achieve this, 10 mL of Luria-Bertani (LB) medium was inoculated with 5 μL of an *E. coli* K12 glycerol stock in a 50 mL falcon tube and incubated overnight at 180 rpm and 37 °C. The next day, the OD_600nm_ of the ONC was measured and adjusted to an OD_600nm_ of 0.75 with LB medium. 75 μL of the adjusted ONC were added to each well of a flat bottom 96-well plate except for 4 wells that only contained LB medium as a blank. After the addition of 75 μL of samples to the respective ONC wells, the plates were incubated for 120 min (80 rpm, 37 °C). Subsequently, the OD_600nm_ was measured with a SpectraMaxPlus384 microplate reader (Molecular Devices, San Jose, CA, USA). For determination of bacterial growth via colony counting, ONCs of *E. coli* K12 were adjusted to an OD_600nm_ of 1.0 with LB medium. Then, 50 µL of bacteria was mixed with 50 µL of supernatant and incubated for 2.5 h at 37 °C in a thermomixer at 300 rpm. Bacteria were subsequently plated on LB agar plates in 1 to 10,000 dilutions, incubated overnight at 37 °C, and colonies were counted manually.

### 4.12. Glucose Measurement and Analysis of the Warburg Effect

Glucose concentrations in the harvested cell culture supernatants 72 h post-stimulation were determined using the Glucose (GO) Assay Kit (Sigma-Aldrich) according to [3]. The metabolic rate was calculated as the percentage of glucose consumption. For this, the measured glucose concentration in the samples was subtracted from the glucose concentration in the stock media (RPMI1640, 2 mg/mL). The Warburg Effect in culture media was determined as 1/OD570 nm, normalized to unstimulated controls according to [3].

### 4.13. Metabolic Flux Analysis

For metabolic flux analysis, 8 × 10^4^ mDCs per well were seeded in Seahorse XF96 cell culture microplates (V3-PS, TC-treated, Agilent, Santa Clara, CA, USA). The next day, the medium was exchanged and cells were stimulated as indicated with the different inhibitors, proteins, TLR ligands, or controls. Seahorse XF Real-Time ATP rate assays were performed according to the manufacturer’s recommendations (Agilent). Post measurement, samples were normalized to total protein content via BCA (Thermo Fischer Scientific, Dreieich, Germany) and analyzed using Wave Desktop Software (Agilent) and Graphpad Prism v8 (Graphpad Software, Inc, San Diego, CA, USA). ATP rate assays were analyzed using the respective report generator sheet according to the manufacturer’s recommendations (Agilent).

### 4.14. Statistical Analysis

Statistical analysis was performed with GraphPad Prism (GraphPad Prism version 9.2.0 for Windows, GraphPad Software, San Diego, CA, USA) for Windows using two-way ANOVA tests adjusted for multiple comparisons according to either Dunnett’s multiple comparison Test (Figure 6; Repos. Figures S1B, S2B and S5B), uncorrected Fisher’s LSD (Repos. Figure S1C,D), or Tukey’s Test (all other figures). For statistically significant results, the following convention was used: * *p* < 0.05, ** *p* < 0.01, and *** *p* < 0.001.

## 5. Conclusions

In summary, we have shown the metabolic changes in rFlaA:Betv1-stimulated mDCs to significantly contribute to the initiated overall immune responses. We could show the effector function of rFlaA:Betv1-activated mDCs to mainly rely on glycolysis and fatty acid metabolism with both pathways significantly contributing to cytokine secretion, expression of co-stimulatory molecules, the production of antimicrobial molecules, and the mDC-derived modulation of T cell responses. These results not only give us a better understanding of the metabolism of dendritic cells activated by TLR-ligand containing vaccines, but also show that the usage of metabolic inhibitors in activated immune cells can strongly modulate the induced immune responses. Understanding these relationships and identifying potential treatment targets in future will likely improve therapeutic outcomes for both allergen specific immunotherapy (AIT) and other diseases.

## Supplementary Materials

The following supporting information can be downloaded at: https://www.mdpi.com/article/10.3390/ijms232012695/s1.

## Abbreviations

2-DG	2-deoxyglucose.
Acetyl-CoA	Acetyl coenzyme A.
APC	Antigen presenting cell.
BCA	Bicinchoninic acid assay.
BPTES	Bis-2-(5-phenylacetamido-1,3,4-thiadiazol-2-yl)ethyl sulfide.
CPT1a	Carnitine palmitoyltransferase 1.
DBA	Dimethyl-bisphenol A.
ECAR	Extracellular acidification rate.
ELISA	Enzyme-linked immunosorbent assay.
FCCP	Carbonylcyanid-p-trifluoromethoxyphenylhydrazon.
FSL(-1)	Pam2CGDPKHPKSF.
HIF-1α	Hypoxia inducible factor 1 subunit alpha.
IFN	Interferon.
Ig[X]	Immune globulin [X].
IL-[X]	Interleukin [X].
LB	Luria-Bertani.
LPS	Lipopolysaccharide.
MAPK	Mitogen activated protein kinase.
mDC(s)	Myeloid dendritic cell(s).
MPLA	monophosphoryl Lipid A.
mTOR	Mechanistic target of rapamycin.
NFκB	Nuclear factor kappa of activated B cells.
OCR	Oxygen consumption rate.
rBet v 1	Recombinant major birch pollen allergen 1.
rFlaA	Recombinant Flagellin A.
rFlaA:Betv1	Recombinant fusion protein consisting of rFlaA and Bet v 1.
rpm	Revolutions per minute.
ROS	Reactive oxygen species.
TH1/2	T helper cell type ½.
TLR	“Toll” like receptor.
YC-I	5-[1-(Phenylmethyl)-1H-indazol-3-yl]-2-furanmethanol.
