# Supplementary material for: Role of Glycolysis and Fatty Acid Synthesis in the Activation and T Cell-Modulating Potential of Dendritic Cells Stimulated with a TLR5-Ligand Allergen Fusion Protein
Source: Int J Mol Sci. 2022 Oct 21;23(20):12695. doi: 10.3390/ijms232012695 (PMC9604253; doi:10.3390/ijms232012695)
Supplement: Supplementary file 1 [file ijms-23-12695-s001.zip › ijms-1940872-supplementary-done.pdf]

International Journal of
Molecular Sciences

MDPI

Supplementary Figures

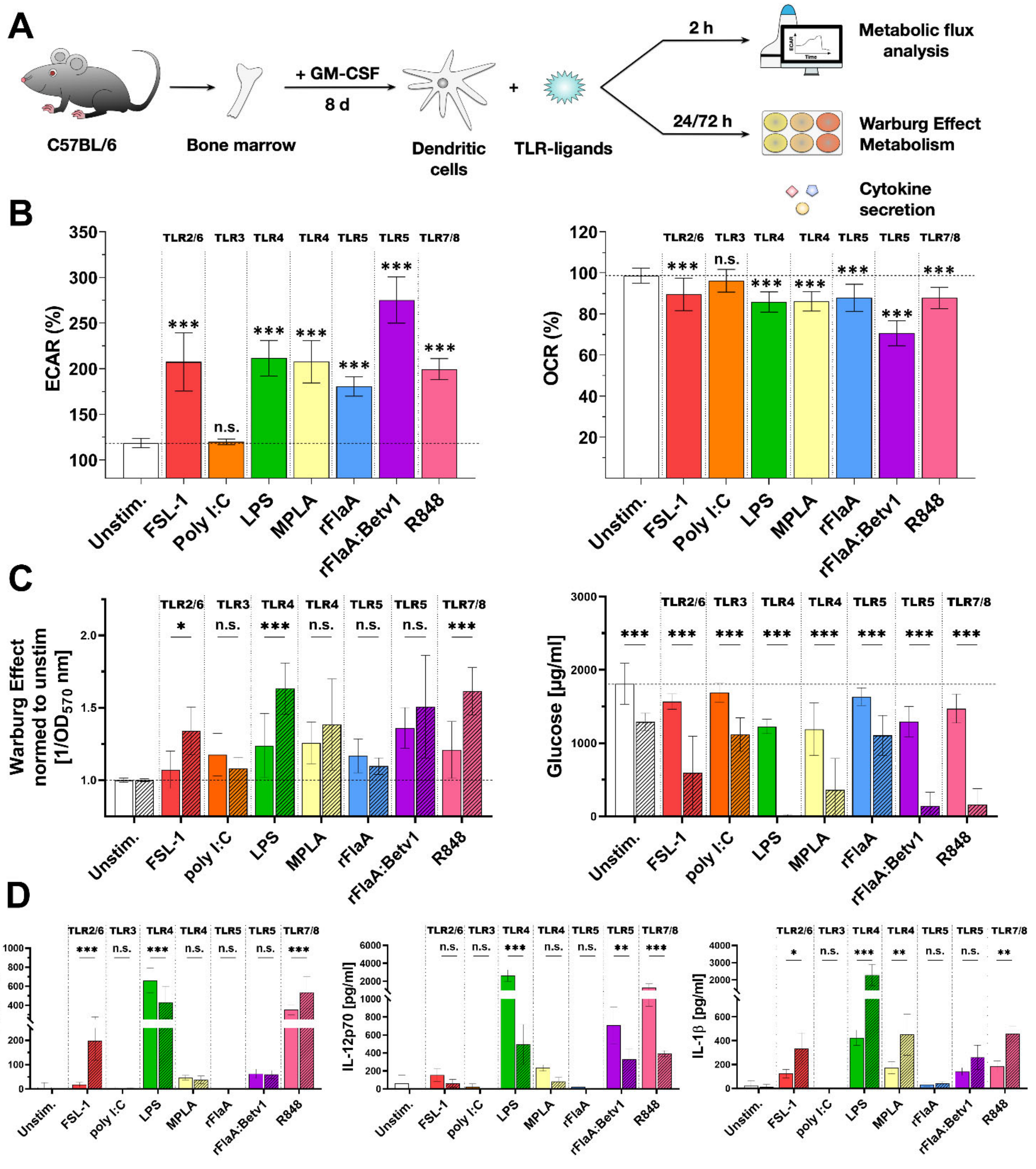

Repository Figure S1: TLR-ligands commonly increase glycolysis while reducing oxidative phosphorylation to varying degrees. Bone marrow-derived dendritic cells (mDCs) were measured using an extracellular flux analyzer for extracellular acidification rates (ECAR) and oxygen consumption rates (OCR) after injection of the indicated TLR-ligands (FSL-1 (TLR2/6-ligand, 0.2 µg/mL), poly I:C (TLR3-ligand, 2 µg/mL), LPS (TLR4-ligand, 2 µg/mL), MPLA (TLR4-ligand, 2 µg/mL), rFlaA (TLR5-ligand, 7 µg/mL), rFlaA:Betv1 (TLR5-ligand, 11 µg/mL), or R848 (TLR7/8-ligand, 0.2 µg/mL)). Measurements were normalized to total protein content by BCA protein quantification before data analysis (**A**). Extracellular acidification rates (ECAR) and oxygen consumption rates (OCR) were calculated after 18 cycles (108 min) (**B**). mDCs were stimulated with the different TLR-ligands (FSL-1

(TLR2/6-ligand, 1 µg/mL), poly I:C (TLR3-ligand, 10 µg/mL), LPS (TLR4-ligand, 10 µg/mL), MPLA (TLR4-ligand, 10 µg/mL), rFlaA (TLR5-ligand 17.4 µg/mL), rFlaA:Betv1 (TLR5-ligand, 27.4 µg/mL), or R848 (TLR7/8-ligand, 1 µg/mL)) and analyzed for the activation of metabolism (Warburg Effect and glucose consumption) (**C**) and cytokine secretion 24 (solid bars) and 72 h (bars with striped pattern) post-stimulation (**D**). Data are mean results of three independent experiments±SD. Statistics were calculated in comparison to the respective unstimulated control samples (**B**) or as indicated (**C, D**) by either 2-way ANOVA with correction for multiple comparisons according to Dunnett (**B**) or uncorrected Fisher's LSD (**C, D**). Statistical significances are indicated as: n.s. p-value > 0.05, * p-value < 0.05, ** p-value < 0.01, or *** p-value < 0.001.

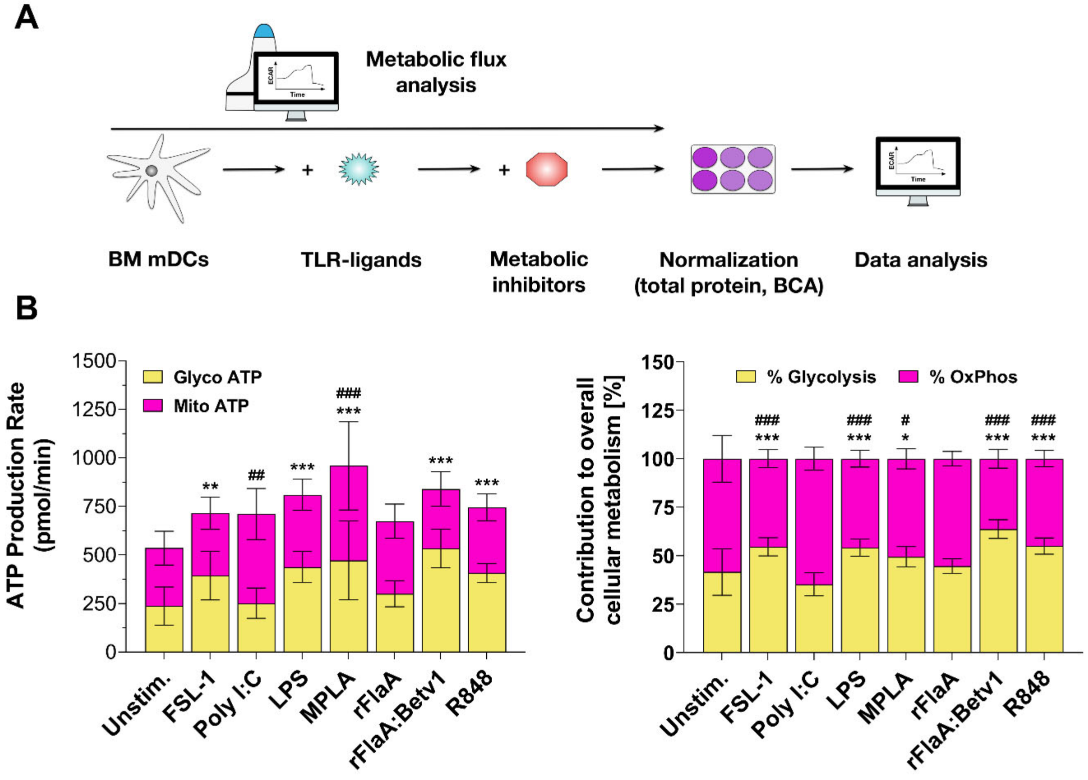

Repository Figure S2: TLR-ligands commonly increase glycolytic ATP production in mDCs. mDCs were stimulated with the indicated TLR-ligands (FSL-1 (TLR2/6-ligand, 0.2 µg/mL), poly I:C (TLR3-ligand, 2 µg/mL), LPS (TLR4-ligand, 2 µg/mL), MPLA (TLR4-ligand, 2 µg/mL), rFlaA (TLR5-ligand 7 µg/mL), rFlaA:Betv1 (TLR5-ligand, 11 µg/mL), or R848 (TLR7/8-ligand, 0.2 µg/mL)) and analyzed using a Seahorse XFe96 Metabolic Flux Analyzer. Measurements were normalized to total protein content by BCA protein quantification before data analysis (**A**). Cells were analyzed for either glycolytic (Glyco ATP) or mitochondrial ATP production rates (Mito ATP) (**B, left**) as well as % contribution of either glycolysis or oxidative phosphorylation (OxPhos) to the overall cellular metabolism (**B, right**) using the Agilent ATP Rate assay and the corresponding report generator sheet. Data are representative results of three independent experiments±SD. Statistics were calculated in comparison to the respective unstimulated control samples by 2-way ANOVA with correction for multiple comparisons according to Dunnett (**B**). Statistical significances are indicated for Glyco ATP or % Glycolysis as: * p-value < 0.05, ** p-value < 0.01, *** p-value < 0.001, or for either Mito ATP or % OxPhos as # p-value < 0.05, ## p-value < 0.01, ### p-value < 0.001, respectively. If not indicated otherwise, no statistical significance was reached (p-value > 0.05).

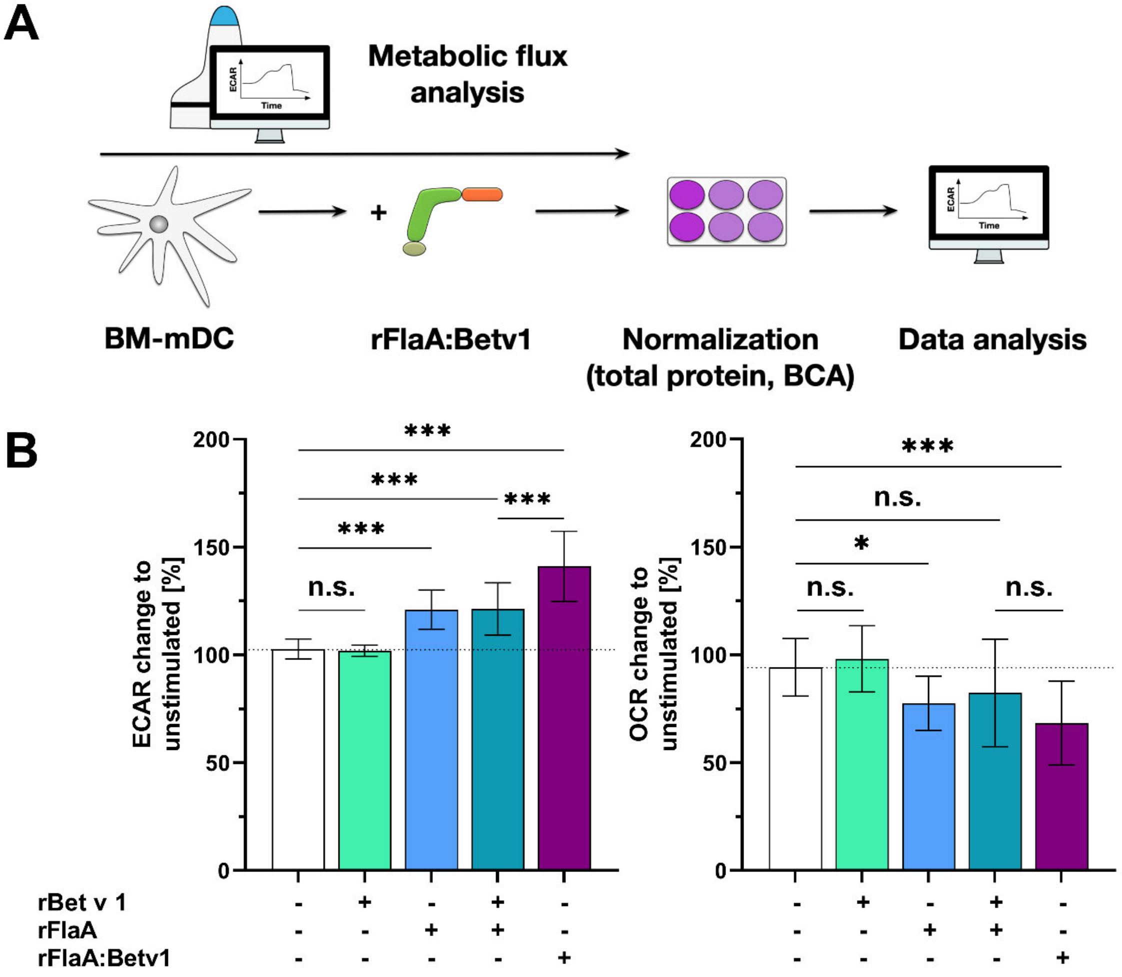

Repository Figure S3: Influence of rFlaA:Betv1 and respective controls on extracellular acidification and oxygen consumption. C57Bl/6J mDCs were left unstimulated for 4 cycles (20 min, defined as 100% after cycle 4) and subsequently stimulated with equimolar concentrations of either rFlaA + rBet v 1 or rFlaA:Betv1 (equimolar to 4 µg/mL of rBet v 1) or 100 ng/mL LPS as a positive control and analyzed for extracellular acidification rates (ECAR) and oxygen consumption rates (OCR) using Seahorse technology (**A**). 14 cycles (84 min) post-stimulation, cells were analyzed for changes in either ECAR (**B, left**) or OCR (**B, right**). Data are mean results of four independent experiments±SD. Statistical comparisons were performed by 2-way ANOVA with correction for multiple comparisons according to Turkey and indicated as: n.s. p-value > 0.05, * p-value < 0.05, ** p-value < 0.01, or *** p-value < 0.001 (B).

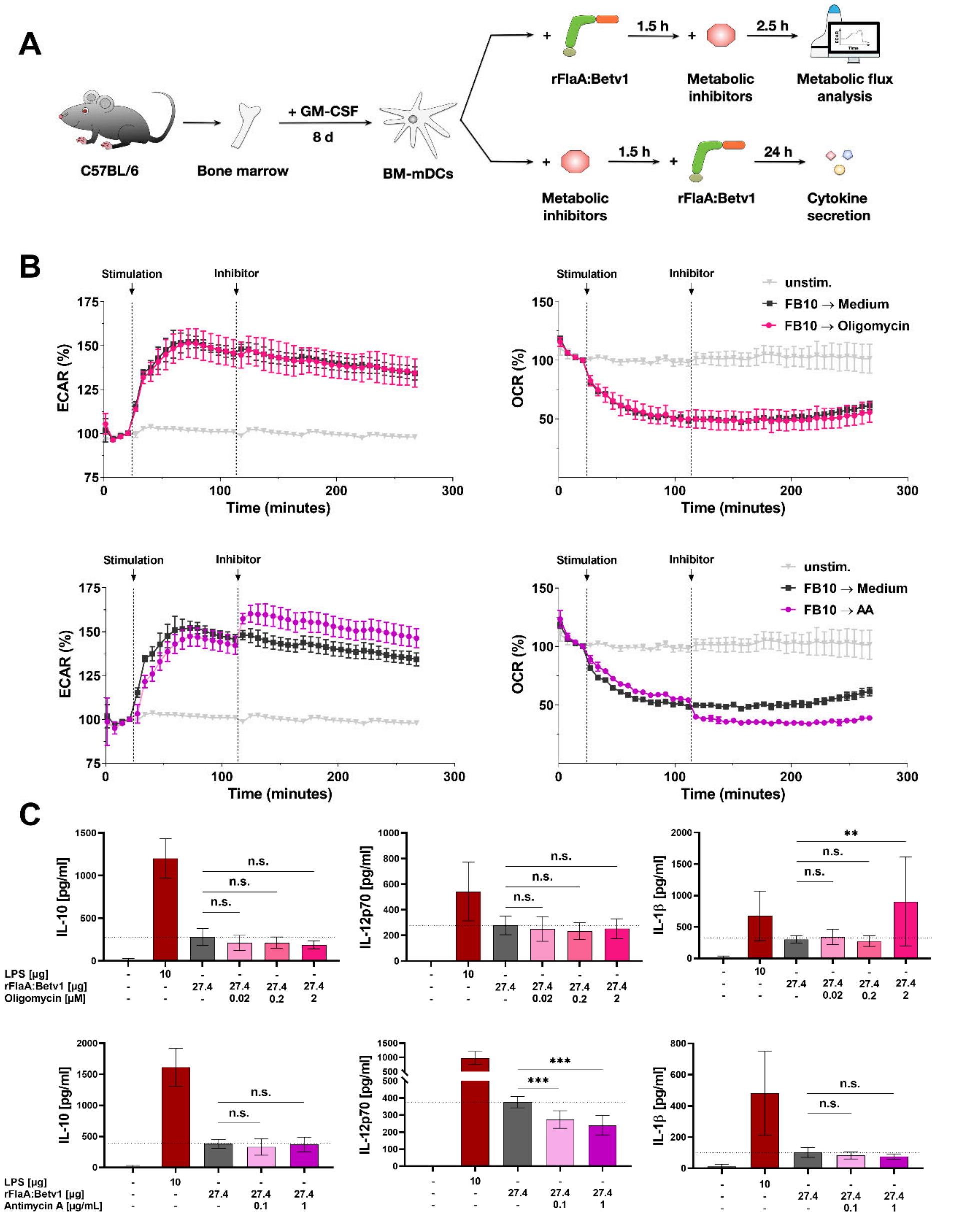

Repository Figure S4: Mitochondrial respiration does neither contribute to rFlaA:Betv1-induced metabolic activation nor cytokine secretion. mDCs were stimulated with 11 µg/mL rFlaA:Betv1 (FB 10) for 14 cycles (84 min) followed by injection of either oligomycin (1.5 µM) or antimycin A (AA, 0.5 µM) for another 24 cycles (144 min) and continuously analyzed for extracellular acidification rates (ECAR) and oxygen consumption rates (OCR) using Seahorse technology (A, B). mDCs were pre-treated for 90 minutes with the indicated inhibitor concentrations followed by stimulation with 27.4 µg/mL of rFlaA:Betv1 for 24 hours. Supernatants were analyzed for cytokine secretion by ELISA (C). Data are either representative results of three to four independent experiments that showed similar results (B, with three to four technical replicates per experiment) or results of three to four independent experiments (C). Statistical comparisons were performed by 2-way ANOVA with correction for multiple comparisons according to Turkey and indicated as: n.s. p-value > 0.05, * p-value < 0.05, ** p-value < 0.01, or *** p-value < 0.001.

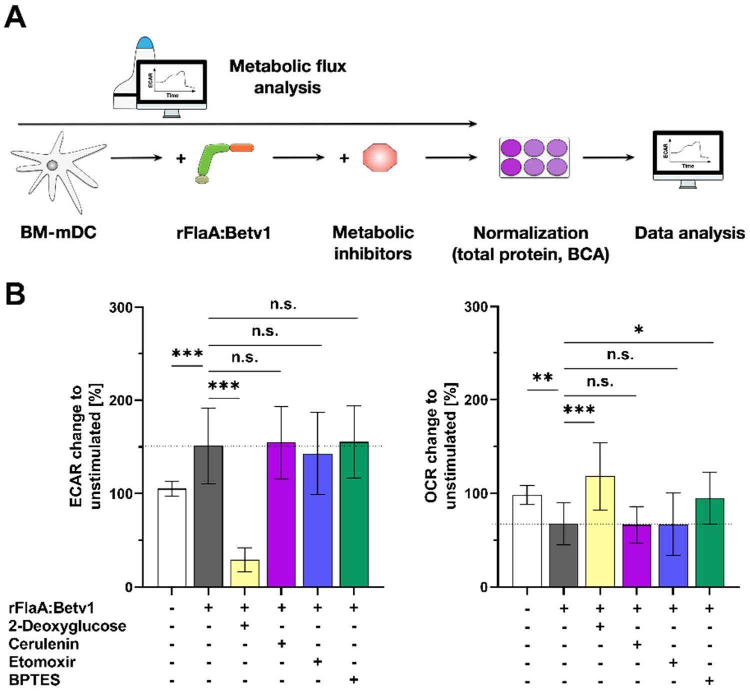

Repository Figure S5: Influence of the used metabolic inhibitors on rFlaA:Betv1-induced changes in ECAR and OCR. mDCs were left unstimulated for 4 cycles (20 min, defined as 100%) and subsequently stimulated with 11 µg/mL rFlaA:Betv1 for 14 cycles (84 min), followed by injection of the different inhibitors (2-DG: 50 mM, cerulenin: 2 µg/mL, etomoxir: 100 µM, BPTES: 20 µM) for another 24 cycles (144 min) and continuously analyzed for extracellular acidification rates (ECAR) and oxygen consumption rates (OCR) using Seahorse technology. Measurements were normalized to total protein content by BCA protein quantification before data analysis (**A**). Cells were analyzed for changes in either ECAR (**B, left**) or OCR (**B, right**) 7 cycles post-treatment (45 min) with the respective inhibitors. Data are mean results of four independent experiments±SD. Statistical comparisons were performed by 2-way ANOVA with correction for multiple comparisons according to Dunnett and indicated as: n.s. p-value > 0.05, * p-value < 0.05, ** p-value < 0.01, or *** p-value < 0.001 (**B**).

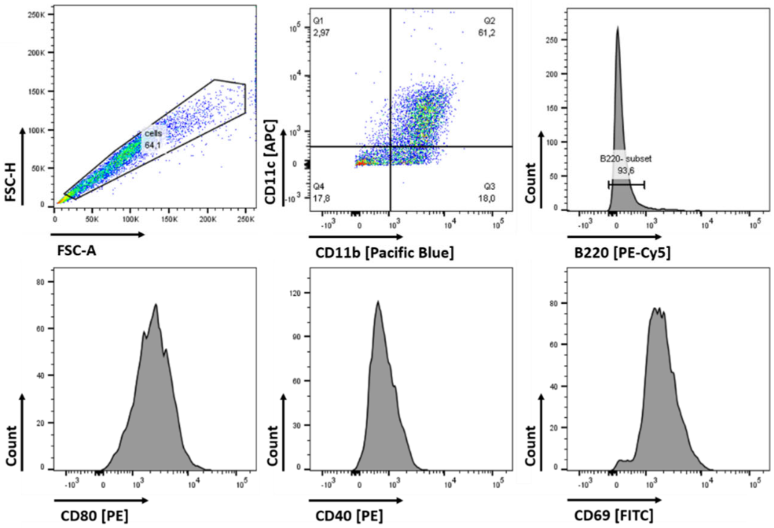

Repository Figure S6: Gating strategy to elucidate the expression of co-stimulatory molecules on mDCs. mDCs differentiated for 8 days in the presence of GM-CSF were seeded in 24 well plates and left unstimulated for 24 hours. On the next day, cells were harvested, stained with the indicated

antibodies, and single cells were discriminated by FSC-H vs. FSC-A. CD11b^{+}CD11c^{+} double positive cells were checked for B220 expression. B220 negative cells were analyzed for surface expression of CD80, CD40, and CD69 in two separate stainings using FlowJo software. Data are representative results of three independent experiments that showed similar results.

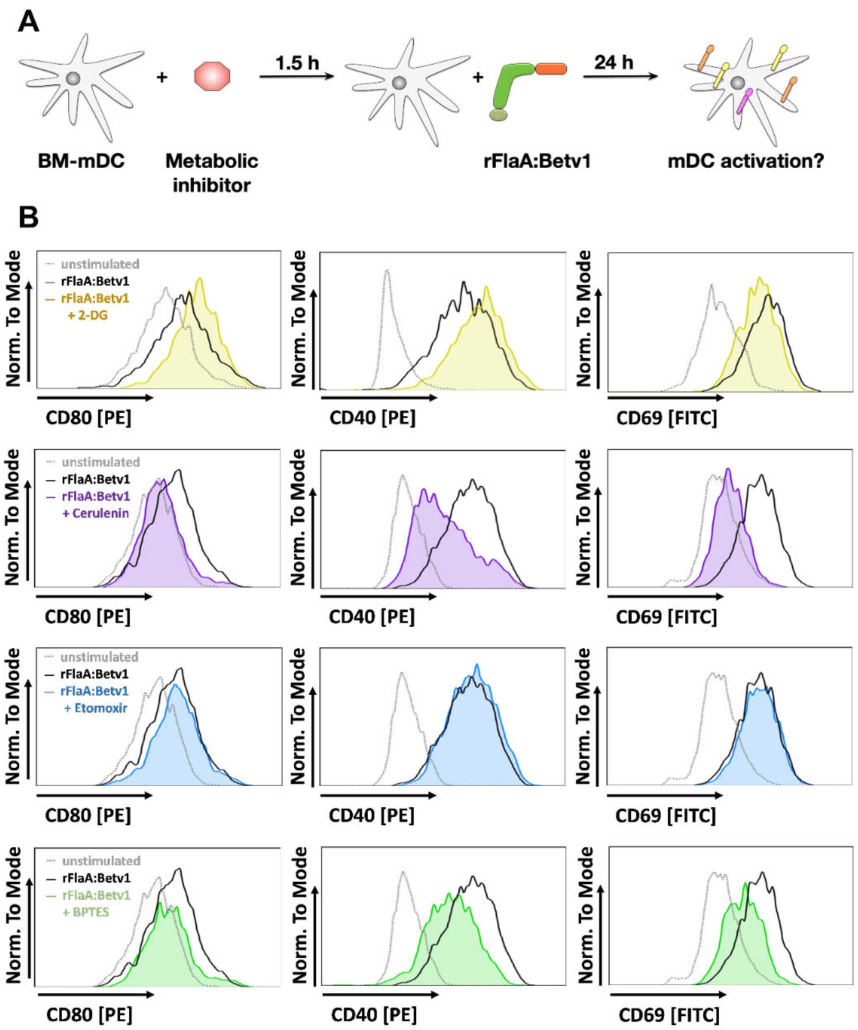

Repository Figure S7: Glycolysis, fatty acid synthesis, and amino acid metabolism contribute to rFlaA:Betv1-induced expression of co-stimulatory molecules. mDCs were pre-treated for 90 minutes with the following inhibitor concentrations: 1 mM 2-DG; 2 µg/mL Cerulinin; 10 µM Etomoxir; 20 µM BPTES. Subsequently, cells were stimulated with 27.4 µg/mL of rFlaA:Betv1 for 24 hours (A). CD11b^{+}CD11c^{+}B220^{-} cells were analyzed for surface expression of the indicated co-stimulatory molecules by flow cytometry (B). Data are representative results of three independent experiments that showed similar results. Please note, that data shown in Fig. 4 and Fig. 5 were generated using the same mDC preparations.

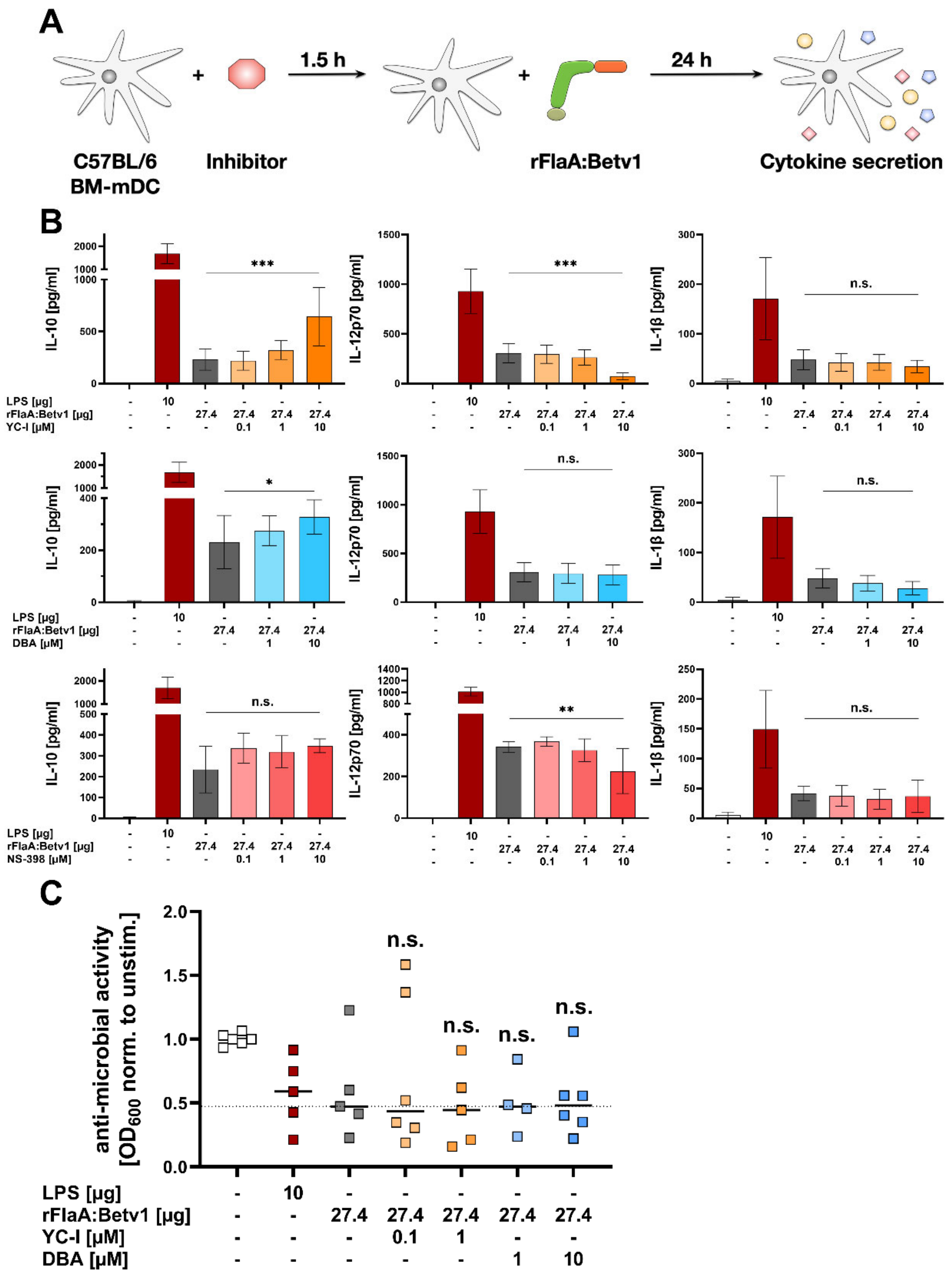

Repository Figure S8: HIF-1α contributes to rFlaA:Betv1-induced IL-10 secretion but not anti-microbial activity. mDCs were pre-treated for 90 minutes with the indicated concentrations of either the HIF-1α-inhibitors YC-I or DBA or the COX2-inhibitor NS-398 followed by stimulation with 27.4 µg/mL of rFlaA:Betv1 for 24 hours (**A**). Supernatants were analyzed for cytokine secretion by ELISA (**B**) and their anti-microbial activity (normalized to unstimulated mDC supernatants, **C**). Statistics were either calculated as indicated (**B**) or in comparison rFlaA:Betv1-stimulated samples (**C**). Data are mean results of three independent experiments. Statistical comparisons were performed by 2-way ANOVA with correction for multiple comparisons according to Turkey and indicated as: n.s. p-value > 0.05, * p-value < 0.05, ** p-value < 0.01, or *** p-value < 0.001.
